# Effect of *Cichorium glandulosum* on intestinal microbiota and bile acid metabolism in db/db mice

**DOI:** 10.1002/fsn3.3694

**Published:** 2023-10-25

**Authors:** Junlin Yan, Rui Zhang, Jinsen Kang, Yewei Zhong, Adalaiti Abudurexiti, Huiwen Tan, Yi Lei, Xiaoli Ma

**Affiliations:** ^1^ College of Pharmacy Xinjiang Medical University Xinjiang China

**Keywords:** bile acids, *Chorum glandulosum* Boiss. et Huet, intestinal microbiota

## Abstract

This study aims to investigate the effects of *Chorum glandulosum* Boiss. et Huet (CG) on the intestinal microbiota and serum bile acid (BA) in db/db mice. A total of 12 db/db mice were randomly divided into model (MOD), high‐dose CG (CGH), and control (CON) groups. The CON and MOD groups received distilled water by gavage for 8 weeks. Whereas, the CGH group received an alcohol extract of CG at a dose of 200 mg/kg/day. Results showed that CG can reduce blood lipid levels. It change the composition of the intestinal microbiota, and increase the relative abundances of Muribaculaceae, Prevotellaceae, *Bifidobacterium_pseudolongum*, Bacteroidaceae in db/db mice as well. LC–MS metabolomics results showed that CG adjusted the serum BA levels. The results reduced the levels of primary BAs, such as cholic acid (CA) and chenodeoxycholic acid (CDCA). The results decreased the primary BA/secondary BA (PSA/SBA) ratio in db/db mice. Correlation analysis showed that the abundances of *Bifidobacterium_pseudolongum* and Bacteroidaceae were positively correlated with acetic acid level and negatively correlated with ursocholic acid (UCA), α‐muricholic acid (αMCA), triglyceride (TG), and total cholesterol levels (TC), indicating an interaction between the intestinal microbiota and serum BAs. CG may play a positive role in the interaction between the intestinal microbiota and BAs in lipid metabolism.

## Graphical abstract

Chicory affects T2DM by affecting the liver–intestinal axis.

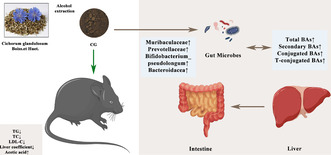

## 1 INTRODUCTION

Owing to economic developments and lifestyle changes, type 2 diabetes mellitus (T2DM) has become one of the most dangerous chronic diseases. In China, the prevalence of diabetes is as high as 11.2% (Chinese Medical Association, Diabetes Branch, 2021), and approximately 40%–50% of diabetic patients have lipid metabolism abnormalities (Li et al., 2020), which can increase the risk of cardiovascular diseases. The intestinal microbiota is considered a key environmental factor in the development of metabolic diseases mainly due to its important role in the regulation of various processes, including host energy metabolism, intestinal peptide hormone secretion, and host inflammatory status (Sangeeta & Mirza, 2020; Sonnenburg & Backhed, 2016). In addition, the structure and composition of the intestinal microbiota of patients with T2DM are altered compared with those of normal individuals; for example, the abundance of beneficial and harmful bacteria decreases and increases, respectively, in patients with T2DM (Larsen et al., 2010). Therefore, treatments that regulate the structure of the intestinal microbiota to alleviate metabolic diseases, including T2DM, have attracted considerable interest.

Bile acids, which are synthesized by the liver, maintain the balance of lipid metabolism in the body, transport fat in the intestine, and promote fat absorption (Yang & Zhang, 2020). Primary bile acids are directly synthesized in the liver from cholesterol as the substrate and can be further converted into secondary bile acids through the deconjugation and dehydroxylation of the intestinal microbiota (Fiorucci et al., 2021; Funabashi et al., 2020). The administration of antibiotics that alter the intestinal microbiota can alter bile acid fraction, inhibit ileal FGF15 gene expression, and upregulate hepatic CYP7A1 expression and hepatic bile acid synthesis in mice (Bäckhed et al., 2005). The interaction between the intestinal microbiota and bile acids mediates host glycolipid level and energy metabolism and is a promising strategy for the treatment of metabolic diseases.

Active ingredients in herbal medicines can alleviate high‐fat‐diet‐induced obesity and metabolic disorders by affecting the relationship between the intestinal microbiota and bile acids or other metabolites, such as short‐chain fatty acids, and have good effects (Liu et al., 2022). *Cichorium glandulosum Boiss*. et Huet. or chicory (CG) belongs to the *Cichorum* genus of the family Asteraceae, and its aboveground parts, roots, and seeds are used as food or medicine (National Pharmacopoeia Committee, 2020). It mainly contains terpenoids, flavonoids, phenolic acids, polysaccharides, and other chemical components. Sesquiterpenes, such as lactucin and lactupicrin, are the main active components of CG. The Xinjiang Herbal Medicine Handbook (Ministry of Health, Logistics Department, Xinjiang Army, 1970) states that CG clears heat and can be used as a detoxifying agent, diuretic, or decongestant. In addition, CG has significant hypoglycemic and lipid‐reducing effects, alleviates multiple metabolic disorders, and improves pharmacological effects, showing clear benefits for the treatment of metabolic diseases (Perović et al., 2021). CG extracts can reduce blood glucose and lipid levels and alleviate liver lipid deposition in T2DM–NAFLD SD rats (Qin et al., 2019). Our previous research showed that CG lowers glucose and lipid levels and thus reduces insulin resistance and changes the structure of the intestinal microbiota (Yan et al., 2022). However, the mechanism by which CG regulates the body's metabolism and microbial diversity needs to be further investigated.

In this study, we investigated the effects of CG on the intestinal microbiota and serum bile acids in db/db mice by establishing a T2DM db/db mouse model with metabolic abnormalities. Then, we explored the associations among blood lipids, the intestinal microbiota, and bile acids in db/db mice through 16srRNA sequencing and LC–MS bile acid‐targeted metabolomics. Our results provided novel insights into the role of CG in microbial homeostasis.

## 2 MATERIALS AND METHODS

### 2.1 Materials and animals

The herb was procured from Mo Yu County, Hotan, Xinjiang, and identified by Prof. Hu Jun Ping of the Department of TCM, Faculty of Pharmacy, Xinjiang Medical University, as *Cichorum glandulosum Boiss*. et Huet. The alcoholic extract of *Cichorum glandulosum Boiss*. et Huet was obtained by weighing and crushing the whole herb of *Cichorum glandulosum Boiss*. et Huet, adding six times the amount of 75% ethanol, overnight heating and extracting on the next day, and concentrating the filtrate. Twelve male SPF‐ db/db mice at 6 weeks of age and 6 male SPF‐ m/m mice at 6 weeks of age in the normal group Animal Production and Use License: SCXK (Su) 2016‐0010 were purchased from Changzhou Cavins Laboratory Animal Co. and housed in the SPF‐grade animal room of the Animal Experiment Center of Xinjiang Medical University. The experiemental conditions were: constant room temperature (25 ± 1)°C, relative humidity (55–65)%, and daily light‐and‐dark cycle 12 h/12 h. All experimental protocols of this study were approved by the Animal Ethics Committee of Xinjiang Medical University, with the ethical approval number IACUC‐20210422‐07.

### 2.2 Animal administration and grouping

The db/db mice were randomly divided into model groups (db/db + Vehicle, MOD group) and *Cichorum glandulosum Boiss*. et Huet alcohol extract administration group (db/db + CG 200 mg/kg, CGH group). Mice in the normal group (m/m + Vehicle, CON group) were given an equal volume of distilled water by gavage for 8 weeks. After the last administration, mice were fasted overnight and fasting blood glucose (FBG) in tail vein blood was measured the next day using a glucometer ACCU‐CHEK Performa at week 8. Livers were removed, weighed, and stored in liquid nitrogen at −80°C for storage. Fresh fecal samples from each animal at week 8 were collected in a clean fume hood, placed in sterile centrifuge tubes, and stored at −80°C. A portion of the sample was used to determine short‐chain fatty acid content by gas chromatography. Blood was taken from the eye and centrifuged at 4°C for 10 min at 13,400 *g* to obtain serum, and a portion of the serum was used to determine total cholesterol (TG), total cholesterol (TC), low density lipoprotein (LDL), and high‐density lipoprotein (HDL) content by biochemical kits. The fecal samples and serum samples were sent to Shanghai Bacitracin Biomedical Technology Co., Ltd., for sequencing of intestinal microbiota and detection of bile acid content. The content of intestinal short‐chain fatty acids such as acetic acid, propionic acid, and butyric acid in the remaining feces was determined by gas chromatography.

### 2.3 Extraction and 16SrRNA sequencing of intestinal microbiota DNA

The DNA extraction of fecal samples was carried out according to the instructions of the Magnetic Soil and Stool DNA Kit (TIANGEN, batch number DP180427). The extracted DNA was detected by 1% agarose gel electrophoresis. CTAB/SDS method is used to extract DNA from the bacterial community in mouse feces. After its quality is qualified, the concentration is tested and the DNA sample is diluted to a concentration of 1 ng/μL. Using specific primers 515F (5′‐GTGCCGCGGTAA‐3′) and 806R (5′‐GGACTACHVGGGTWTCTAAT‐3′) as templates, the bacterial 16S rRNA V3‐V4 region was selected for PCR amplification. The products were purified, quantified, and homogenized to form a sequencing library. The established library was first subjected to library quality inspection, referring to Qiime ([V1.9.1], http://qiime.org/scripts/split_libraries_fastq.html) using NovaSeq6000 for machine sequencing. The original image data file obtained through high‐throughput sequencing is transformed into the original sequencing sequence through base recognition analysis. The sequencing samples were clustered into OTUs using the SILVA database sequence as a reference sequence, and functional annotation information was obtained to draw a relative abundance map of species annotations. Bioinformatics uses the LIMS2 analysis cloud platform for analysis (https://www.lims2.com/). Sequencing and analysis were carried out by Shanghai Bacitracin Biomedical Technology Co.

### 2.4 Detection of bile acids by LC–MS/MS

In view of not having precesion experiemental equipments, we decided to hire a medical analysis company. We picked Shanghai Baiqu Biomedical Technology Co.,Ltd, and sent the materials to them. They detects serous bile acid content based on LC–MS/MS platform. Take 100 μL serum sample in EP tube, add 400 μL extraction solution methanol:acetonitrile (volume ratio 1:1, containing 1% formic acid and 62.5 nmol/L internal standard), vortex for 30 s, mix well, sonicate in an ice water bath for 5 min, let stand at 20°C for 1 h, centrifuge at 4°C and 13,400 *g* for 15 min, and take 75 μL supernatant to injection vial for LC–MS/MS analysis. Mobile phase conditions: Vanquish (Thermo Fisher Scientific) ultrahigh‐performance liquid chromatography using Waters ACQUITY UPLC BEH C18 (150 × 2.1 mm, 1.7 mm) μm. Waters liquid chromatography column is used for the chromatographic separation among the target compound. Phase A of liquid chromatography is 1 mmol/L ammonium acetate and 0.1% acetic acid aqueous solution, and phase B is acetonitrile. The temperature of the column incubator is 50°C, the sample tray is set at 4°C, and the injection volume is 1 μL. Mass spectrometry conditions: Q Active HFX high‐resolution mass spectrometer, using parallel reaction monitoring (PRM) mode for mass spectrometry analysis. The ion source parameters are as follows: spray voltage = +3500/−3100 V, sheath gas (N2) flow rate = 40, aux gas (N2) flow rate = 15, sweep gas (N2) flow rate = 0, aux gas (N2) temperature = 350°C, and capillary temperature = 320°C. The original UPLCMS/MS data are processed using MultiQuant software (version 3.0.3) for peak detection, calibration, and standardization. Statistical analysis was conducted using SPSS 22.0 (IBM) statistical software, and *p* < .05 was considered statistically significant. The original data were processed, and the principal component analysis (PCA) and orthogonal partial least squares discriminant analysis (OPLS‐DA) of the serum bile acid metabolic profile were performed using SIMCA (V15.0.2, Umetrics). After verifying that the original model was suitable for subsequent analysis, the VIP value was calculated using R language, the difference of bile acid was screened out, and the *p* value was determined by the two‐tailed *t*‐test (VIP > 1.0 and *p* < .05).

### 2.5 Statistical analysis

Statistical analysis was performed using SPSS 22.0 software, and the measurement data were expressed as mean ± standard deviation (mean ± SEM), the differences between groups were compared using one‐way ANOVA (one‐way ANOVA), *t*‐test of two independent samples was used for two‐way comparison, and *p* < .05 was considered as a statistically significant difference. The software we applied, GraphPad Prism 8.0 software, was used for graphing. In the experiment, correlations were calculated using the correlation among lipids, short‐chain fatty acids, and serum bile acids, intestinal species and families.Serum bile acids and intestinal species and families were calculated using the spearman correlation analysis. *p* values < .05 were considered statistically significant.

## 3 RESULTS

### 3.1 Effect of CGH on blood lipids and short‐chain fatty acids in db/db mice

**FIGURE 1:**
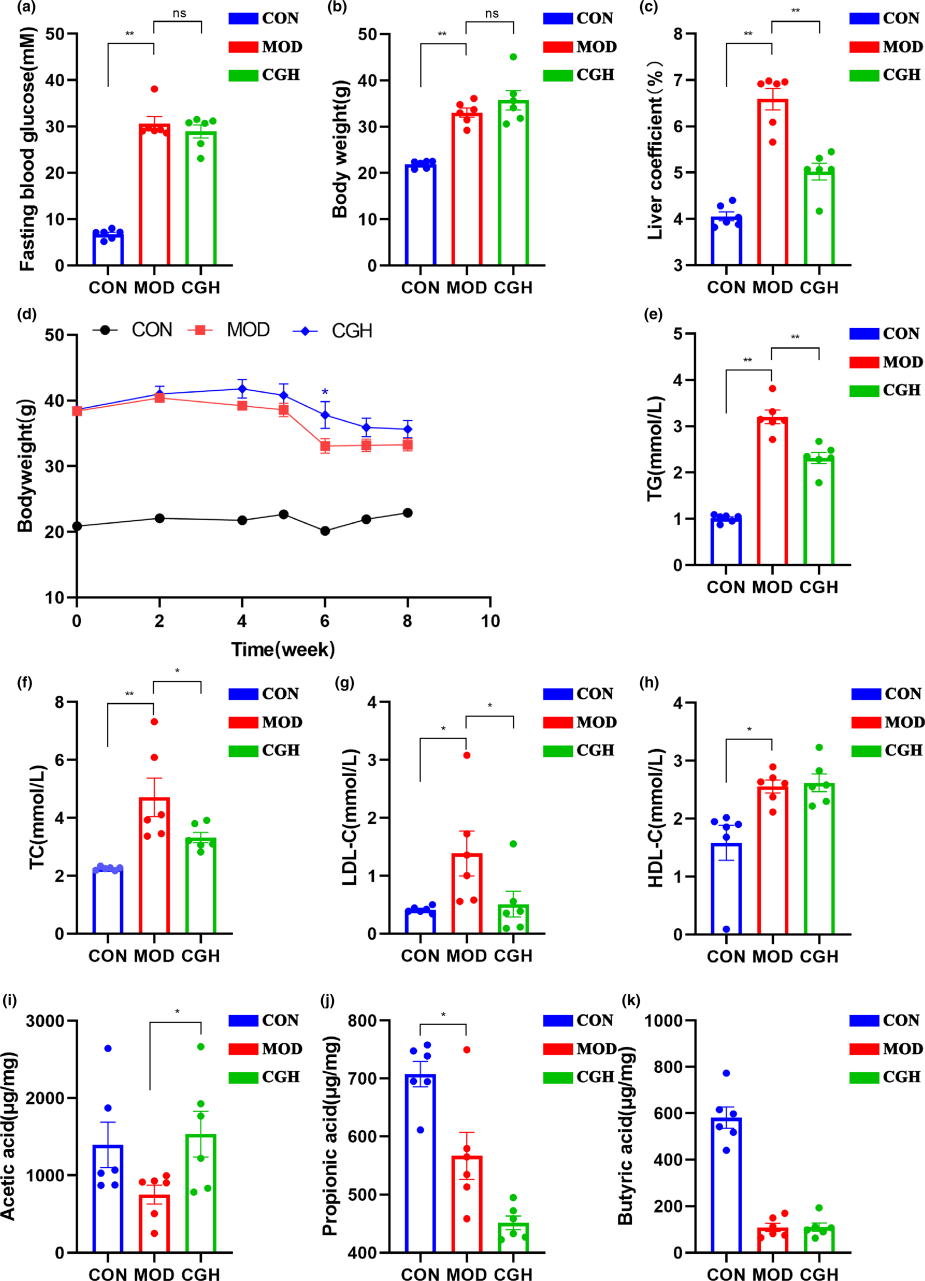
Effect of CGH supplementation on fasting blood glucose at week 8 in db/db mice (a); body weight at week 8 (b); liver organ coefficients (c); weight changes during the experiment (d); TG (e); TC (f); LDL (g); HD (h); acetic acid (i); propionic acid (j); and butyric acid (k). Data are presented as the means ± SEM (*n* = 6), **p < .01, *p < .05, ns, no significant difference.

As shown in Figure 1a,b, the glucose test fasting blood sugar of mice in MOD group at week 8 was significantly higher than that in CON group (*p* < .01). After administration of chicory, the blood glucose and body weight of CGH group mice showed no significant changes compared to the MOD group (Figure 1d). However, the liver coefficient of the MOD group mice was significantly higher than that of the CON group and had a statistical difference (*p* < .01). After administration of chicory, the liver coefficient of the CGH group mice was significantly lower than that of the MOD group (*p* < .01; Figure 1c). As shown in Figure 1e–h, compared with the CON group mice, the serum TC, TG, and LDL levels in the MOD group mice were significantly increased (*p* < .01), while the serum TC, TG, and LDL levels in the CGH group were significantly decreased compared to the MOD group, with statistical differences (*p* < .01 or *p* < .05). The HDL content showed an upward trend compared to the MOD group, but there was no significant difference. In addition, Figure 1h–j represent the levels of three SCFAs in the feces of db/db mice, with the CON group significantly higher than the MOD group (*p* < .01; Figure 1i–k); The acetic acid content in the CGH group significantly increased (*p* < .05).

### 3.2 Effect of CGH on intestinal microbiota of db/db mice

**FIGURE 2:**
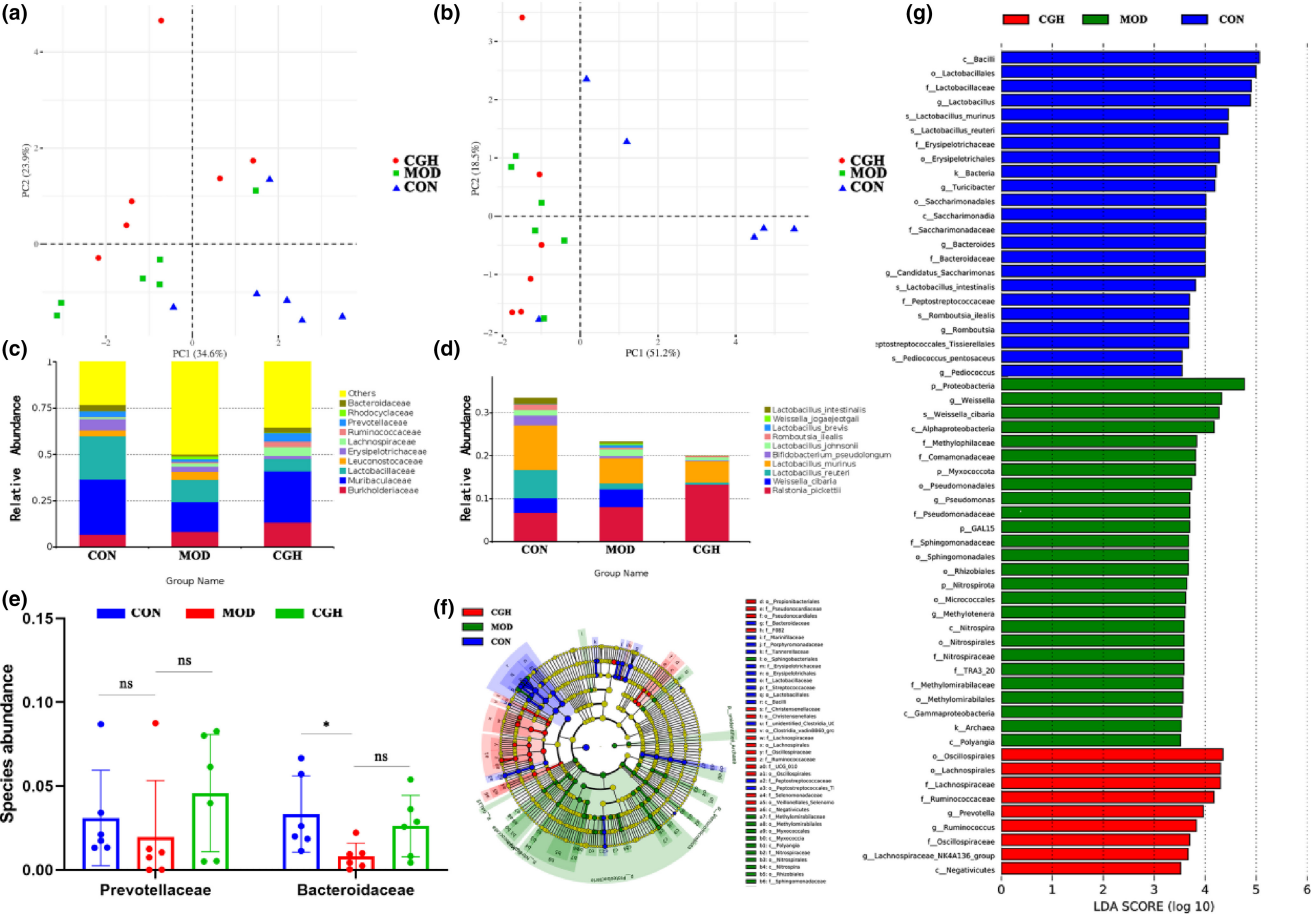
Effect of CGH on intestinal microbiota of db/db mice. Principal coordinate analysis (PCA) based on weighted (relative abundance) UniFrac distances of intestinal microbiota of normal and db/db mice family (a); species (b); relative abundance of intestinal microbiota at family level (c); relative abundance of intestinal microbiota at species level (d); effect of CGH on intestinal Prevotellaceae and Bacteroidaceae (e); and Lefse analysis at family and species level and LDA scores above 3 (f, g). Data are presented as the means ± SEM (*n* = 6), **p < .01, *p < .05, ns, no significant difference.

The intestinal microbiota structure of the three groups of mice was analyzed using 16SrRNA gene amplicon sequences, the differences in the intestinal microbiota of mice were analyzed by PCA method at the level of family and species, and the results are shown in figure (Figure 2a,b). The distribution of the microbiota of mice in the CGH and CON groups was more aggregated, while there was a clear separation between the microbiota of mice in the MOD group and the microbiota of mice in the CON group. This suggests that CGH has a significant effect on the intestinal microbiota of db/db mice. Based on the species annotation results, the top 10 species in terms of maximum abundance at the family and species level for each sample were selected to generate a cumulative bar chart of species' relative abundance in order to visualize the species with higher relative abundance and their proportions at different taxonomic levels for each sample. At the family level, the most abundant groups were Muribaculaceae, Lactobacillaceae, and Burkholderiaceae, where the MOD group compared to the CON group of mice, Muribaculaceae, Lactobacillaceae, Erysipelotrichaceae, Prevotellaceae, and Bacteroidaceae decreased in abundance, and Muribaculaceae, Prevotellaceae, Lactobacillaceae, and Bacteroidaceae increased in abundance in the CGH group compared to the MOD group. At the species level, *Ralstonia_pickettii* and *Lactobacillus_murinus* were the dominant species, the abundance of *Weissella_cibaria* increased in the MOD group compared to the CON group, and the abundance of *Weissella_cibaria* decreased in the CGH group compared to the MOD group. The abundance of *Ralstonia_pickettii* increased in the CGH group compared to the MOD group (Figure 2c–e). The results of LefSe analysis indicated significant differences in the distribution of microbiota in each group of mice at the phylum‐to‐species level. The significantly enriched communities in the CON group of mice included Bacilli, Lactobacillales, Lactobacillaceae, and *Lactobacillus* The significantly enriched communities in the MOD group included Proteobacteria, *Weissella*, *Weissella_cibaria*, Alphaproteobacteria, etc. The significantly enriched communities in the CGH group included Oscillospirales, Lachnospirales, Lachnospiraceae, Ruminococcaceae, etc. (Figure 2f,g).

### 3.3 Effect of CGH on serum bile acids in db/db mice

**FIGURE 3:**
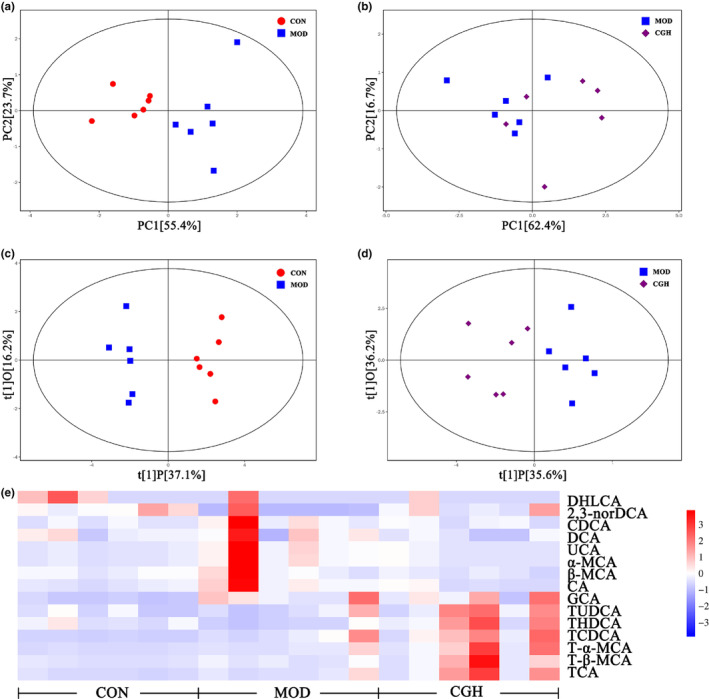
Effect of CGH on the composition and ratio of bile acids. PCA score plot for CON and MOD groups (a); PCA score plot for MOD and CGH groups (b); OPLS‐DA score plot for CON and MOD groups (c); OPLS‐DA score plot for MOD and CGH groups (d); and heatmap of 15 bile acids in serum (e).

**FIGURE 4:**
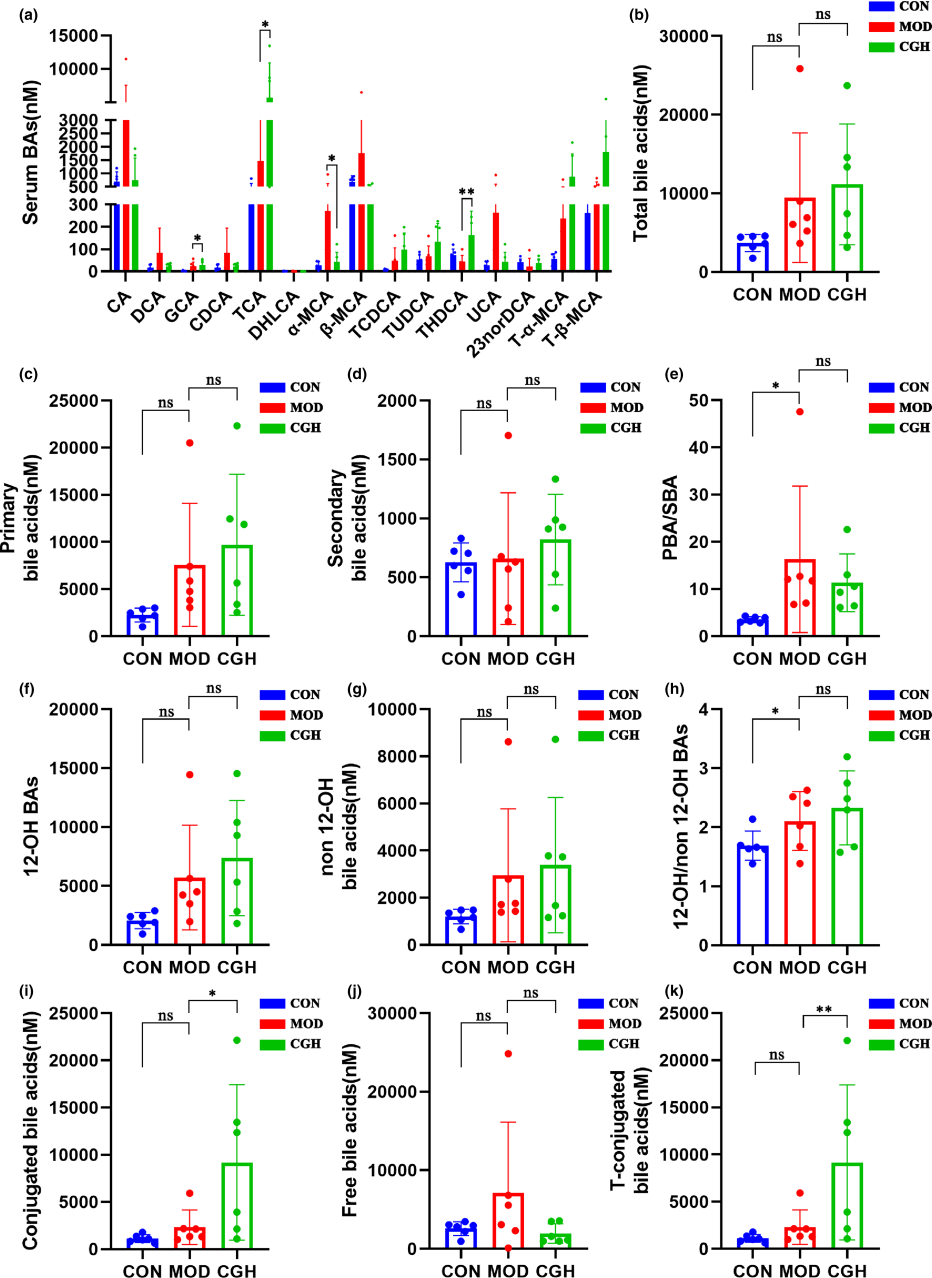
Serum bile acid content in three groups. Data are presented as the means ± SEM (*n* = 6), **p < .01, *p < .05, ns, no significant difference.

The pictures illustrate the effect of CGH on serum bile acid levels in db/db mice as shown in Figures 3 and 4. The PCA score plots show a significant separation between the CON and MOD groups (Figure 3a) and the MOD and CGH groups (Figure 3b). According to the orthogonal partial least‐squares discriminant analysis (OPLS‐DA) model, score plot shows a significant separation between the CON and MOD groups (Figure 3c) and the MOD and CGH groups (Figure 3d). Figure 3e is a heatmap of 15 bile acids in the serum of 3 groups of mice. As shown in Figure 4, the contents of deoxycholic acid (DCA), **α‐**muricholic acid (αMCA), β‐muricholic acid (β‐MCA), taurochenodeoxycholic acid (TCDCA), ursocholic acid (UCA), 23‐nordeoxycholic acid (23norDCA), tauro‐α‐muricholic acid (T‐α‐MCA), and tauro‐β‐muricholic acid (T‐β‐MCA) increased and taurohyodeoxycholic acid (THDCA) decreased in the MOD group compared to the CON group (Figure 4a). Compared with the CON group, the MOD group had an increasing trend of primary bile acid content (Figure 4c) and a significant increase in PBA/SBA (*p <* .05) (Figure 4e). The CGH group had an increasing trend of T‐binding bile acid content (*p* < .01) compared with the MOD group (Figure 4k), an increasing trend of secondary bile acid content (Figure 4d) and a decrease in PBA/SBA (Figure 4e). The proportion of non‐12α‐OH bile acids such as TUDCA, T‐α‐MCA, and T‐β‐MCA increased in the CGH group (Figure 4a), where 12α‐OH bile acids/non 12α‐OH bile acids were negatively correlated with insulin resistance, and 12α‐OH/non 12α‐OH decreased in the CGH group after administration (Figure 4f–h)., indicating that the CGH group db/db mice's insulin resistance was alleviated to some extent. In addition, compared to the MOD group, the content of conjugated bile acids in the CGH group significantly increased or decreased, and the content of T‐conjugated bile acids was also significantly higher than that in the MOD group. The content of free bile acids was slightly higher in the MOD group, but there was no significant difference (Figure 4i–k).

The *t*‐test test was performed for 29 bile acids, and the variable projection importance (VIP) of the first principal component of the OPLS‐DA model was calculated using R language, and VIP > 1 and *p*‐value < 1 were used as screening conditions to screen the differential bile acids in the model group and normal group, and the model group and chicory group, and the screening results are shown in Tables 1 and 2 below. Through the screening of VIP > 1 and *p*‐value < 1, the model A total of seven differential bile acids were screened in the MOD and CON groups, including TCDCA, isolithocholic acid (isoLCA), hyocholic acid (HCA), 23norDCA, tauroursodeoxycholic acid (TDCA), β‐MCA, and hyodeoxycholic acid (HDCA), of which TCDCA was increased in the model group and the rest of the bile acids were decreased compared with the model group (Table 1). A total of four species of differential bile acids were screened in the CGH group and the MOD group, including T‐β‐MCA, taurolithocholic acid (TLCA), tauroursodeoxycholic acid (TUDCA), and THDCA, among which T‐β‐MCA decreased in the administration group compared with the model group, and the rest showed an increasing trend (Table 2).

**TABLE 1:** Differential bile acids screening table between model (MOD) and control (CON) groups.

MS2 name	MOD group	CON group	VIP	*p*‐Value	Fold change	LOG_Foldchange
TCDCA	0.0060 ± 0.0046	0.0024 ± 0.0012	1.04	.06	2.53	1.34
isoLCA	0.0002 ± 0.0001	0.0007 ± 0.0006	1.65	.05	0.22	−2.17
HCA	0.0004 ± 0.0003	0.009 ± 0.0005	1.32	.04	0.49	−1.03
23norDCA	0.0011 ± 0.0006	0.0105 ± 0.0066	1.39	.01	0.10	−3.29
TDCA	0.0374 ± 0.0211	0.0734 ± 0.0219	1.34	.01	0.51	−0.97
β‐MCA	0.0684 ± 0.0699	0.1794 ± 0.0335	1.32	.002	0.38	−1.39
HDCA	0.0061 ± 0.0042	0.0229 ± 0.0064	1.43	.00004	0.27	−1.90

**TABLE 2:** Differential bile acids screening table between high dose *Chorum glandulosum* Boiss. et Huet (CGH) and model (MOD) groups.

MS2 name	CGH group	MOD group	VIP	*p*‐Value	Fold change	LOG_Foldchange
T‐β‐MCA	0.0610 ± 0.0658	0.0846 ± 0.0636	1.61	.49	0.72	−0.47
TLCA	0.0003 ± 0.0002	0.0002 ± 0.0001	1.42	.16	1.85	0.89
TUDCA	0.0403 ± 0.0363	0.0109 ± 0.0052	1.77	.08	3.70	1.89
THDCA	0.0484 ± 0.0471	0.0087 ± 0.0029	1.91	.07	5.56	2.47

### 3.4 Correlation analysis

**FIGURE 5:**
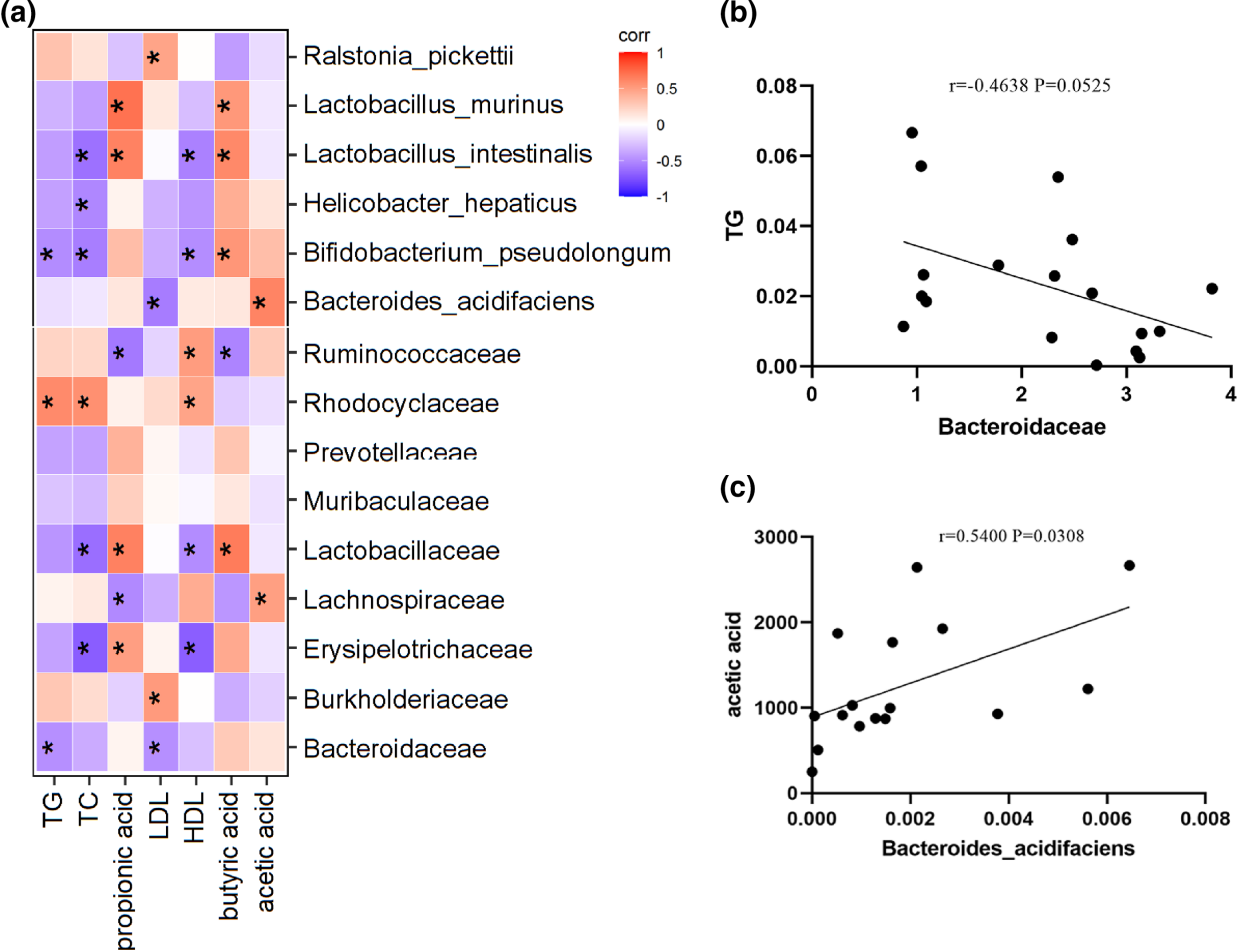
Correlation analysis among blood lipids, short‐chain fatty acids, and intestinal microbiota in mice; the red degree indicates that the relationship between them tends to be positive; on the contrary, the blue degree indicates that the relationship between them tends to be negative and asterisk indicates *p* < .05. (a); scatter plot between Bacteroidaceae and TG (b); *Bacteroides_ acidifaciens* with acetic acid (c). Scatter plots were used to visualize the relationship between different enterobacteria and other indicators.

The results of Spearman's correlation analysis of intestinal microbiota with blood lipids and short‐chain fatty acids in mice feces are shown in Figure 5a. The abundance of *Helicobacter_hepaticus* and *Romboutsia_ilealis* was negatively correlated with TG and TC, and the abundance of Rhodocyclaceae was negatively correlated with TG and TC. Figure 5b shows a significant positive correlation between TG and Bacteroidaceae (*r* = .4638, *p* = .0525), and acetic acid and *Bacteroides_ acidifaciens* (*r* = .5400, *p* = .0308).

**FIGURE 6:**
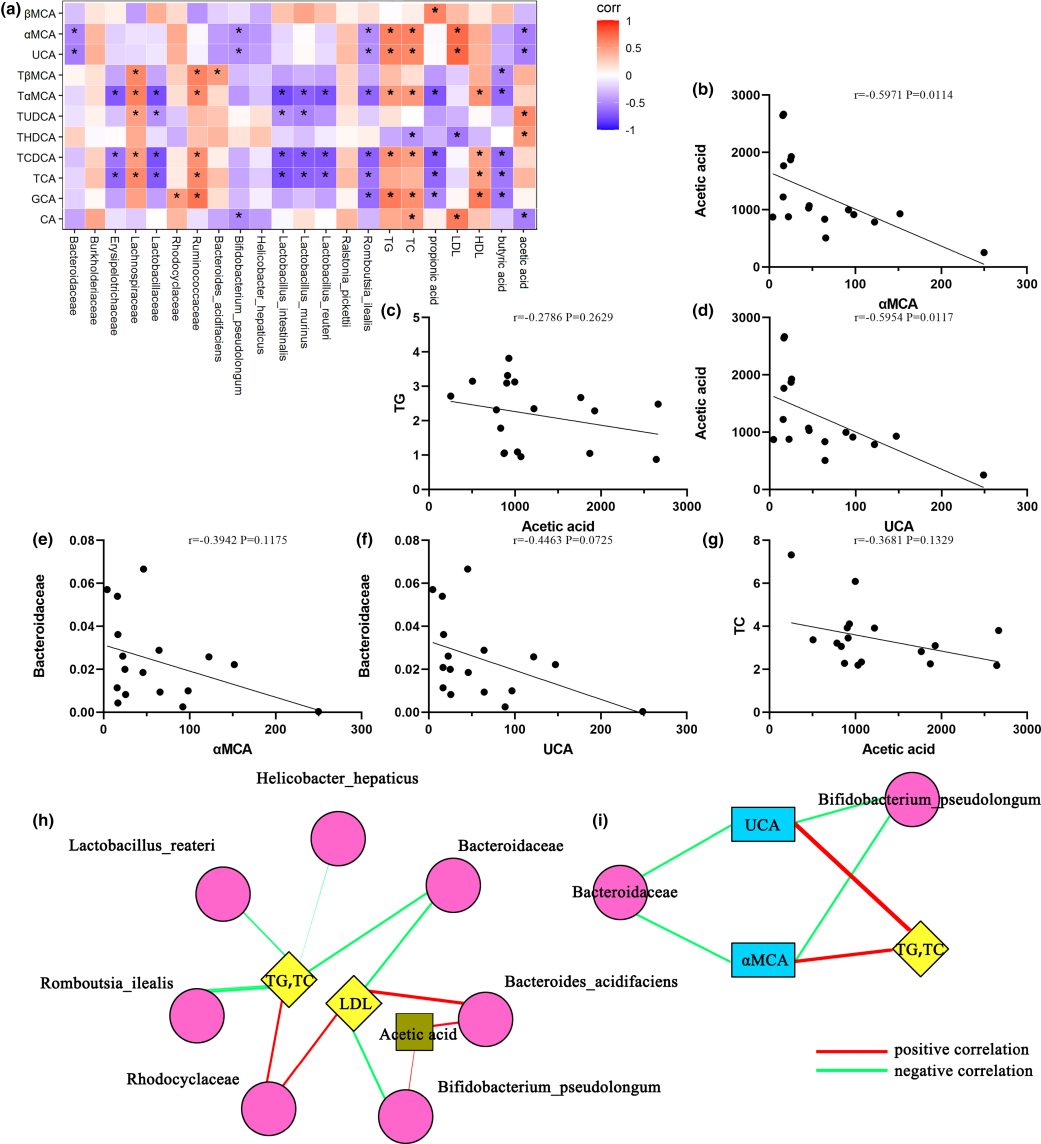
Correlation analysis among blood lipids, short‐chain fatty acids, intestinal microbiota, and serum bile acids in mice; the red degree indicates that the relationship between them tends to be positive; on the contrary, the blue degree indicates that the relationship between them tends to be negative, and asterisks indicate *p* < .05. (a); Scatter plot between αMCA and acetic acid (b); scatter plot between acetic acid and TG (c); scatter plot between UCA and acetic acid (d); scatter plot between αMCA and Bacteroidaceae (e); scatter plot between UCA and Bacteroidaceae (f); scatter plot between acetic acid and TC (g). (The scatter plots were used to visualize the relationship between serum bile acids and other indicators at different enterobacterial levels). These two graphs are a visual display of the correlation between different gut microbiota and blood lipids, SCFAs, and bile acids in db/db mice (h, i) (The thicker the line, the more significant the correlation).

The results of Spearman correlation analysis between serum bile acids and fecal intestinal microbiota, blood lipids, and short‐chain fatty acids in mice are shown in Figure 6a. Among them, αMCA and UCA were closely correlated with several intestinal microbiota, blood lipids, and short‐chain fatty acids. When the content of αMCA and UCA increased, the abundance of *Bacteroidaceae, Bifidobacterium_pseudolongum, and Romboutsia_ilealis* would decrease correspondingly, the content of acetic acid would decrease correspondingly, and the content of TG, TC, and LDL would increase correspondingly. Figure 6b,d shows a significant positive correlation between acetic acid and αMCA (*r* = .5971, *p* = .0114) and UCA (*r* = .5954, *p* = .0117).

## 4 DISCUSSION

*Chorum glandulosum* Boiss. et Huet is widely cultivated in southern Xinjiang, China. Modern pharmacological studies have shown that CG mainly contains sesquiterpenes, coumarins, flavonoids, and other chemical components (Jian et al., 2020; Luo et al., 2019; Yang et al., 2020). CG has significant pharmacological effects and potential effect that alleviate hypoglycemia, hypolipidemia, and various metabolism‐related diseases (Li et al., 2018; Singh et al., 2017; Yang et al., 2012). It can lower blood glucose and lipids, alleviate insulin resistance, and affect the structure of the intestinal bacteria in db/db mice, but whether the mechanism by which CG improves the body's metabolism is related to intestinal microbiota and related interconnected processes has not been fully elucidated.

The db/db mouse is a common animal model for T2DM and can be used in studying the physiological and biochemical indicators of obesity and drug therapies. Owing to mutations in the leptin receptor gene, db/db mice lack response to satiety substances. Thus, the degree of anabolism is greater than that of catabolism in these mice. This condition leads to fat accumulation and obesity, and severe T2DM gradually develops (Wua et al., 2013). In this experiment, we observed the efficacy of the 200 mg/kg ethanol extract of CG on db/db mice for 8 weeks. We found that CG reduced the liver coefficient, lowered total cholesterol (TC), triglyceride (TG), and low‐density lipoprotein levels, and alleviated the disorder of lipid metabolism to some extent.

As the largest and most important microecosystem of the organism, the gut microbiota is dependent on its host, and alterations in its structure and diversity induce a range of metabolic diseases, including obesity and diabetes, through metabolic, inflammatory, and immune pathways (Gu et al., 2017; Patterson et al., 2016). The resulting altered metabolites then migrate from the intestine through the compromised intestinal barrier and affect metabolic organs, such as the liver and adipose tissue (Sebastián Domingo & Sánchez Sánchez, 2018).

Chronic metabolic diseases are associated with changes in microbial characteristics, such as elevated Firmicutes/Bacteroides ratio, reduced microbial diversity, and altered microbial function (Hung et al., 2021). Some intestinal microorganisms, such as Bifidobacterium, potentially maintain intestinal barrier function, limit inflammation, and are inversely associated with metabolic diseases (Lim & Shin, 2020). Bacteroides and Prevotella can produce short‐chain fatty acids, such as acetate, propionate, and butyrate, through the anaerobic fermentation of dietary fiber, resistant starch, and undigested proteins (Yu et al., 2019; Zafar & Saier, 2021). As key bacterial metabolites produced by the intestinal microbiota in the colon, short‐chain fatty acids play an important role in the metabolism of substances, maintenance of barrier function, regulation of angiogenesis, maintenance of immune homeostasis, and prevention of pathological environments. They maintain the acidic environment of the intestine, inhibit the growth of harmful bacteria (Akhtar et al., 2021; O'Riordan et al., 2022; Xu et al., 2022), and bind to G‐protein‐coupled receptor 43 to stimulate L cells to regulate energy utilization (Bisenieks et al., 2021). To further investigate the effect of CG on the intestinal microbiota of db/db mice and the metabolites of short‐chain fatty acids, we sequenced the fecal intestinal bacteria of db/db mice by using a 16SrRNA amplicon sequencing technology and detected the content of short‐chain fatty acids in the feces of the db/db mice through gas chromatography. The results showed that supplementation with CG increased the relative abundances of key bacterial groups. The abundances of Muribaculaceae, Prevotellaceae, *Bifidobacterium_pseudolongum*, and Bacteroidaceae decreased in the model (MOD) group compared with those in the control (CON) group, and after treatment with CG, the CGH group showed a decrease in the content of Muribaculaceae compared with that in the MOD group. In the high‐dose CG (CGH) group, the abundance of Muribaculaceae, Prevotellaceae, *Bifidobacterium_pseudolongum*, and Bacteroidaceae increased compared with those in the MOD group. Moreover, compared with the CON group, the MOD group showed decreased acetic acid content, and the acetic acid content of mice in the CGH group increased significantly after the administration of CG. Muribaculaceae belongs to the order Bacteroidaceae of the phylum Bacteroides. It plays an important role in energy metabolism and regulation of blood glucose and blood lipids in the intestine, and bacteria within this family are regarded as beneficial (Smith et al., 2021). The function of Prevotellaceae is associated with the ability to rapidly ferment complex polysaccharides, and the main fermentation products are acetic and succinic acids and small amounts of isobutyric, isovaleric, and lactic acids, which can play a key role in the prevention of obesity and improvement of glucose tolerance (Rampelli et al., 2015). *Bifidobacterium_pseudolongum* belongs to the genus *Bifidobacterium* and can radically improve metabolic disorders by synthesizing nutrients and promoting protein absorption in hosts (Satti et al., 2021). In addition, db/db mice cannot feel satiety because of hypothalamic defects. Acetate can induce the activation of hypothalamic neurons and thus suppresses appetite, reduces exogenous lipid intake, and thus exerts a hypolipidemic effect in db/db mice (Hussain & Krishnamurthy, 2018; Palumbo et al., 2018). The present experiment confirmed that the number of key intestinal microbiota in the db/db mice changed after the administration of CG, the number of beneficial bacteria was upregulated, and the content of acetic acid increased significantly. These results indicated that CG can improve the structure of the intestinal microbiota, increase the content of short‐chain fatty acids, and contribute to the treatment of lipid metabolism disorders.

A mutual communication between the gut microbiota and intestinal lipid metabolism has been discovered, and rats with impaired microbiota exhibit impaired intestinal lipid absorption, celiac particle production, and reduced mucosal apolipoproteins after antibiotic treatment (Bohan et al., 2019), in addition to functional correlation of intestinal microbial metabolism, such as short‐chain fatty acid synthesis, with the intestinal microbiota. In the present study, we found a close association between intestinal microbiota and lipids and short‐chain fatty acids in db/db mice through Spearman correlation analysis. The results showed that the levels of Bacteroidaceae, *Helicobacter_hepaticus, Lactobacillus_reateri*, and *Romboutsia_ilealis* were negatively correlated with TG and TC levels, and the abundances of *Bacteroides_acidifaciens* and *Bifidobacterium_pseudolongum* were positively correlated with acetic acid content. These results suggest a correlation between the intestinal microbiota and lipid, and short‐chain fatty acid production in db/db mice and CG can further affect lipid metabolism and short‐chain fatty acid production by acting on the intestinal bacteria in db/db mice.

Bile acids, as important signals and regulators of bidirectional communication between intestinal microbes and hosts, can perform key pathophysiological functions along the intestine–hepatic axis through a variety of host receptors (Guzman et al., 2020). Moreover, bile acid metabolism is related to lipid metabolism, and lipogenic enzymes in the small intestine can be activated by bile acids to form lipases and bind to TGs, which finally catalyze the digestion and utilization of lipids (Schoeler & Caesar, 2019). Primary bile acids are initially synthesized in the liver and subsequently bound to taurine. In the presence of the intestinal microbiota, bound bile acid is broken down and metabolized into secondary bile acids, which further affect the activity of farnesol X receptor (FXR) and related signaling pathways (Chiang & Ferrell, 2018; Schoeler & Caesar, 2019). Elevated bile acid synthesis rate and 12α‐OH bile acid (CA/DCA)/non‐12α‐OH bile acid ratio were observed in patients with insulin resistance combined with disorders of lipid metabolism (Pathak et al., 2018). The results of this experiment showed an increase in total bile acids in the MOD group compared with the CON group, and total bile acids in the CGH group returned to the level of the CON group after CG administration. Owing to the increases in cholic acid and chenodeoxycholic acid levels, the overall levels of primary bile acids and primary/secondary bile acid (PBA/SBA) ratio decreased. After intervention with CG, the ratio of bile acids in the serum of mice in the CGH group changed considerably, and the levels of secondary, conjugated, taurine‐conjugated, and 12α‐OH bile acids increased compared with those in the MOD group, and the ratios of PBA/SBA and 12α‐OH /non‐12α‐OH bile acids decreased compared with those in the MOD group. We found that the levels of serum bile acids of the db/db mice were almost equal to those of the CON group after intervention with CG; that is, supplementation with CG can influence changes in bile acids in metabolically abnormal db/db mice and adjust serum bile acid levels to the standard levels of normal mice. These results suggest that changing the bile acid profile may be an effective way to treat the metabolic abnormalities of db/db mice.

In the intestine, metabolites produced through diverse microbial metabolic processes alter host‐specific responses. For example, the interaction of the intestinal microbiota with bile acids plays a key role in the regulation of host metabolism (Molinaro et al., 2018). Intestinal metabolite‐sensing mechanisms are mainly determined by host G‐protein‐coupled receptors (GPRs), which play a key role in linking the gut microbiota–metabolite axis; short‐chain fatty acid receptors, such as GPR41 and GPR4 (Fernandes et al., 2014; Ornelas et al., 2022; Park et al., 2021); and the goose deoxycholic acid receptor FXR, which is expressed in enterocytes and other tissues. FXR is associated with the regulation of various host‐specific metabolic processes, particularly inhibiting insulin secretion, reducing nonesterified fatty acid release, inhibiting bile acid synthesis, and regulating cholesterol and triacylglycerol synthesis (Huang et al., 2019; Wang et al., 2018). By investigating the relationship between the intestinal microbiota and serum bile acids, we found that the probiotic Bacteroidaceae, which is negatively correlated with serum lipids in mice, was negatively correlated with *Bifidobacterium_pseudolongum* and α‐ratiocholic acid (αMCA) and ursolic acid (UCA). The αMCA and UCA levels were elevated in the sera of mice in the MOD group compared with the CON group, and the αMCA and UCA levels in the sera of mice in the CGH group decreased after the administration of CG. By contrast, the levels of αMCA and UCA were positively correlated with serum TC and TG levels in mice. *Bifidobacterium_pseudolongum* can regulate the intestinal microbiota of high‐fat‐fed mice and thus reduce triglyceride levels (Bo et al., 2020), and *Bifidobacterium_pseudolongum* belongs to Bifidobacterium and cause changes in serum bile acids and their metabolic parameters in patients with T2DM (Zhu et al., 2021). The elevated levels of Bacteroidaceae increase the ratio of secondary‐to‐primary bile acids, significantly reducing FXR and TGR5 deficiency (He et al., 2021), and a negative correlation between Bacteroidaceae levels and obesity was found (Ye et al., 2021). In this study, we found an interaction between Bacteroidaceae/*Bifidobacterium_pseudolongum* and free bile acids αMCA and UCA, which form a network that regulates lipid and short‐chain fatty acid levels. CG plays an important role in this process, providing an intervention strategy for the treatment of obesity and related metabolic diseases.

*Chorum glandulosum* Boiss. et Huet alleviated dyslipidemia and altered the abundances of key intestinal microbes in the db/db mice, elevating the content of probiotics, such as *Bifidobacterium_pseudolongum* and Bacteroidaceae. It alleviated metabolic dysfunction by altering the serum bile acid profile. We found a close relationship between key microbiota and serum bile acids in the db/db mice, and by identifying the correlations among the intestinal microbiota, short‐chain fatty acids, bile acids, and blood lipids, we identified a potential mechanism by which CG treats abnormal metabolic diseases: intestinal microbiota modulates short‐chain fatty acids and serum bile acids and thereby alter abnormal blood lipid metabolism. Moreover, CG exhibited a network effect on the regulation of intestinal microbiota and bile acid metabolism, providing a theoretical basis for the development of the medicinal function of CG in regulating metabolic abnormalities.

## AUTHOR CONTRIBUTIONS

**Junlin Yan:** Data curation (lead); writing – original draft (equal). **Rui Zhang:** Project administration (lead); writing – review and editing (equal). **Jinsen Kang:** Methodology (supporting); validation (equal). **Yewei Zhong:** Formal analysis (equal); validation (equal). **Adalaiti Abudurexiti:** Resources (equal); software (equal). **Huiwen Tan:** Investigation (equal); supervision (equal). **Yi Lei:** Methodology (supporting); writing – review and editing (supporting). **Xiaoli Ma:** Funding acquisition (lead); resources (lead).

## FUNDING INFORMATION

This research was supported by the Xinjiang Uygur Autonomous Region Tianshan Talent Youth Top Talent Project (Grant No. 2022TSYCCX0104) and the Major Special Projects of Xinjiang Uygur Autonomous Region, China (Grant No. 2022A03007‐3).

## CONFLICT OF INTEREST STATEMENT

All authors certify that they have participated sufficiently in the work to take public responsibility for the appropriateness of the experimental design and method, and the collection, analysis, and interpretation of the data. The authors have reviewed the final version of the manuscript and approved it for publication. To the best of our knowledge and belief, this manuscript has not been published in whole or in part nor is it being considered for publication elsewhere.

## INSTITUTIONAL REVIEW BOARD STATEMENT

The animal study protocol was approved by the Ethics Committee of the Animal Experiment, Middle School of Xinjiang Medical University (protocol code: IACUC‐20210422‐07; Approval Date: 22 April 2021).

## Supporting information


Data S1.

## Data Availability

The data presented in the manuscript are available on request from the corresponding author.
